# Altered pubertal timing in 7q11.23 copy number variations and associated genetic mechanisms

**DOI:** 10.1016/j.isci.2024.109113

**Published:** 2024-02-06

**Authors:** Shau-Ming Wei, Michael D. Gregory, Tiffany Nash, Andrea de Abreu e Gouvêa, Carolyn B. Mervis, Katherine M. Cole, Madeline H. Garvey, J. Shane Kippenhan, Daniel P. Eisenberg, Bhaskar Kolachana, Peter J. Schmidt, Karen F. Berman

**Affiliations:** 1 Behavioral Endocrinology Branch, National Institute of Mental Health, Intramural Research Program, National Institutes of Health, Bethesda, MD, USA; 2 Section on Integrative Neuroimaging, Clinical and Translational Neuroscience Branch, National Institute of Mental Health, Intramural Research Program, National Institutes of Health, Bethesda, MD, USA; 3 Neurodevelopmental Sciences Laboratory, Department of Psychological and Brain Sciences, University of Louisville, Louisville, KY, USA; 4 Human Brain Collection Core, National Institute of Mental Health, Intramural Research Program, National Institutes of Health, Bethesda, MD, USA

**Keywords:** Genetics, Human Genetics, Genotyping

## Abstract

Pubertal timing, including age at menarche (AAM), is a heritable trait linked to lifetime health outcomes. Here, we investigate genetic mechanisms underlying AAM by combining genome-wide association study (GWAS) data with investigations of two rare genetic conditions clinically associated with altered AAM: Williams syndrome (WS), a 7q11.23 hemideletion characterized by early puberty; and duplication of the same genes (7q11.23 Duplication syndrome [Dup7]) characterized by delayed puberty. First, we confirm that AAM-derived polygenic scores in typically developing children (TD) explain a modest amount of variance in AAM (R^2^ = 0.09; p = 0.04). Next, we demonstrate that 7q11.23 copy number impacts AAM (WS < TD < Dup7; p = 1.2x10^−8^, η^2^ = 0.45) and pituitary volume (WS < TD < Dup7; p = 3x10^−5^, η_p_^2^ = 0.2) with greater effect sizes. Finally, we relate an AAM-GWAS signal in 7q11.23 to altered expression in postmortem brains of *STAG3L2* (p = 1.7x10^−17^), a gene we also find differentially expressed with 7q11.23 copy number (p = 0.03). Collectively, these data explicate the role of 7q11.23 in pubertal onset, with *STAG3L2* and pituitary development as potential mediators.

## Highlights

• Polygenic scores for age at menarche (AAM) correlate with AAM in developing girls
• 7q11.23 copy number variation significantly relates to AAM and to pituitary volume
• Differential transcription of *STAG3L2* underlies the 7q11.23 CNV-AAM association

## Graphical abstract

**Figure:**
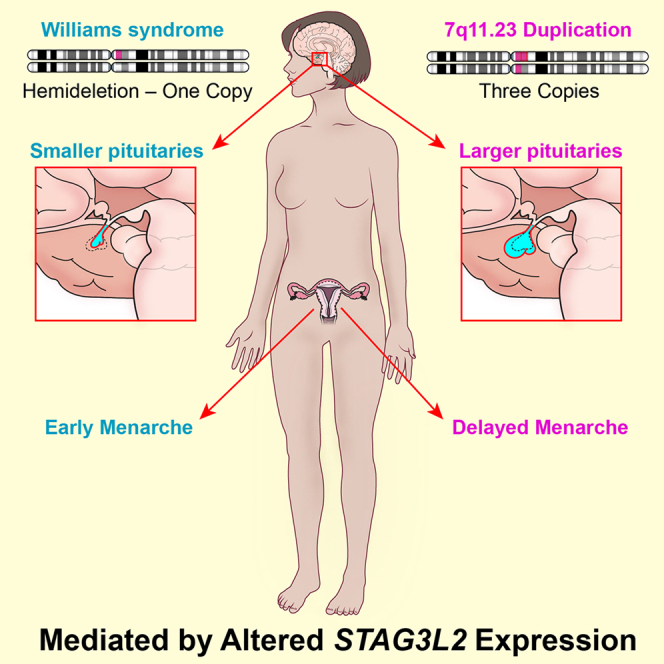

## Introduction

Menarche, when girls begin menstruation, is a pivotal physical developmental milestone that not only holds cultural and social importance in many communities, signifying the onset of reproductive ability, but is also medically meaningful with a range of concurrent and future health implications. Menarche is also a highly important but under-recognized public health consideration. For example, evidence from high-income countries suggests that girls who reach menarche and puberty early without adequate emotional support are likely to engage in earlier sexual relations and substance abuse, posing risks for adolescent pregnancy and other negative health outcomes.^1^^,^^2^^,^^3^^,^^4^

Pubertal maturation of the hypothalamic-pituitary-ovarian axis underlying menarche is complex, and age at menarche (AAM) varies widely across individuals. This variability is associated with subsequent risk for a number of illnesses later in life, including various oncologic, cardio-metabolic, gynecological/obstetric, gastrointestinal, musculoskeletal, and neuropsychiatric diseases.^4^ For instance, early menarche has been associated with increased breast cancer risk,^5^^,^^6^ likely due, at least in part, to longer cumulative estrogen exposure throughout reproductive life.^7^^,^^8^ Additionally, both early and late AAM have been correlated with several metabolic markers that contribute to increased risk of cardiovascular disease; these include high cholesterol, hypertension, and obesity.^9^^,^^10^^,^^11^^,^^12^^,^^13^ Some evidence suggests that, even many decades past menarche, Alzheimer’s disease risk may be elevated in those with later AAM,^14^^,^^15^ though confirmatory work is needed.^16^^,^^17^

Although the timing of puberty may be influenced by a number of environmental and secondary factors, including nutritional status, systemic health, and central nervous system pathology, it is clear that AAM is also genetically driven, showing 50–80% heritability in prior work.^18^^,^^19^^,^^20^^,^^21^^,^^22^ This genetic component raises the possibility that some of the epidemiologic associations between AAM and subsequent disease risk could be mediated not just by the direct sequelae of exposure to a certain cumulative number of lifetime menstrual cycles, but also by underlying pleiotropic genetic variation, though mechanistic evidence from biological experimentation is needed to elaborate this hypothesis. A recent genome-wide association study (GWAS) of ∼370,000 women identified 389 significant independent signals that contribute to AAM,^23^ indicating a highly polygenic architecture guiding this important developmental trait. This complexity is consistent with studies of puberty-related disorders in both human and animal models, which point to a multifaceted network of signaling molecules and pathways regulating hypothalamic-pituitary-gonadal (HPG) maturation.^24^^,^^25^ In addition to the abundance of common, GWAS-defined variants that have been statistically related to normal variability in AAM, investigations into rare highly penetrant genetic mutations, including copy number variants (CNVs), can provide unique opportunities to understand specific molecular drivers of clinical phenotypes,^26^^,^^27^ including pubertal timing abnormalities.

One example of a chromosomal locus where CNVs affect pubertal timing is the Williams syndrome (WS) critical region in the 7q11.23 chromosomal band. Hemideletion of ∼25 genes in this ∼1.5 Mb region causes WS, a rare neurodevelopmental disorder;^28^ compared to typically developing children (TD) who have two copies of these genes and typical age of pubertal onset, it has been clinically noted that children with WS (having one copy of affected genes) may enter puberty early,^29^^,^^30^^,^^31^^,^^32^ or even have precocious puberty.^33^ More recently and in contrast, individuals with duplications of this same chromosomal locus have been identified (7q11.23 duplication syndrome [Dup7], with three copies of these same genes),^34^ and clinical observations by ourselves and others have been suggestive of delayed puberty in these individuals.^35^ If empirically confirmed, these genetically mediated, dose-dependent differences in pubertal onset in 7q11.23 CNVs would provide a unique model for investigating the genetic etiology of variation in the timing of puberty.

In searching for a neural substrate of these genetic findings, we focused on the pituitary gland, a key region in the HPG axis and a central hub for regulating endocrine function in the brain.^36^ Interestingly, data from the GTEx consortium, a large-scale collaborative effort in which DNA and RNA from multiple tissues were sequenced from almost 1,000 deceased individuals,^37^ have shown that the pituitary is enriched with genes underlying the AAM-related variants,^23^ suggesting that it plays a crucial role in the timing of puberty. In TD children, with average pubertal onset and normal endocrine function, pituitary gland volumes in both sexes increase gradually with age, show a growth spurt during early puberty, and are larger in girls than boys.^38^^,^^39^^,^^40^ Studies of pituitary volumes in either precocious or delayed puberty are sparse, with some reports suggesting larger pituitary volumes in children with precocious puberty compared with age-matched controls.^41^^,^^42^ Since 7q11.23 CNVs display differential timing of pubertal onset, we hypothesized that pituitary volumes may also vary across these CNVs, though studies examining this potential neural correlate are lacking.

Here, to better characterize the genetic foundations of AAM, we integrated large-scale, general population statistical findings with in-depth investigations of individuals who have rare 7q11.23 CNVs and are enrolled in our long-term, longitudinal studies. First, we sought to confirm and quantify the contributions of common genetic variation to pubertal timing by relating polygenic scores for AAM to clinician-ascertained AAM in a reference cohort of TD girls. Second, to confirm clinical observations, we directly tested whether and to what degree 7q11.23 CNVs are related to AAM. Next, if pubertal onset is indeed altered by 7q11.23 CNVs, and because there is evidence that pituitary morphology in children with early puberty differs from controls, we also tested for an association between 7q11.23 copy number and pituitary volume. To further pinpoint candidate genes that may be responsible for these associations in WS/Dup7, we then examined the 7q11.23 locus for GWAS-defined AAM-associated single nucleotide polymorphisms (SNPs),^23^ and we further characterized these signals by identification of relevant quantitative trait-loci (eQTLs) and by testing for differential gene expression in an RNASeq analysis of individuals with 7q11.23 CNVs.

## Results

### Polygenic contributions to AAM

**Figure 1:**
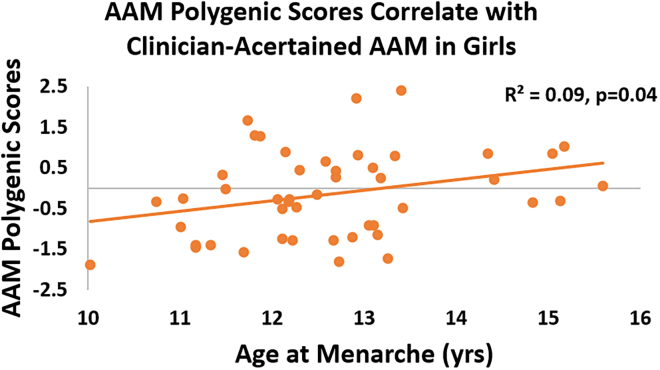
Correlation between PGS-AAM and AAM in typically developing girls PGS-AAM was positively related to clinician-ascertained AAM such that individuals with a polygenic proclivity toward later actual AAM were observed to have a later AAM (R^2^ = 0.09, p = 0.04). Abbreviations: PGS: polygenic scores; AAM: age at menarche.

The degree to which common genetic variation cumulatively influences a phenotype can be estimated as a single value with polygenic scoring methods.^43^ We used these methods, in conjunction with previously reported summary statistics from a GWAS of retrospectively self-reported AAM that included ∼370,000 adult women^23^ in order to confirm and quantify the genetic contribution to pubertal timing in a reference sample of TD girls participating in our longitudinal studies. Based on the GWAS summary statistics for AAM previously reported,^23^ we calculated AAM polygenic scores (PGS-AAM) for 47 TD girls (whose date of first menstruation was recorded soon after it occurred; Table 1). PGS-AAM was positively related to clinician-ascertained AAM such that individuals with a polygenic proclivity toward later AAM were observed to have later actual AAM (r = 0.3, p = 0.04, Figure 1). Common genetic variation accounted for approximately 9% of the variance in AAM within this TD reference group, an effect size consistent with prior reports.^23^

**Table 1:** Demographics

Study	7q11.23 CNVs	Total N	Girls N	Age (mean ± SD)^a^
AAM Polygenic scores in TD girls	WS	N/A	N/A	N/A
	TD	47	47	12.6 ± 1.3
	Dup7	N/A	N/A	N/A
Association of AAM and 7q11.23 CNVs	WS	8	8	10.96 ± 1.1
	Dup7	9	9	15.8 ± 2.7
	TD	47	47	12.6 ± 1.3
	Siblings	16	16	12.45 ± 1.4
Differential Expression Analysis of *STAG3L2* in 7q11.23 CNVs	WS	23	15	9.94 ± 4.0
	TD	40	19	12.86 ± 3.6
	Dup7	13	8	12.17 ± 3.1
Pituitary Volume Size Differences with 7q11.23 CNVs	WS	30	20	11.87 ± 3.9
	TD	50	24	11.88 ± 3.4
	Dup7	16	7	12.51 ± 2.8

a for “AAM polygenic scores in TD girls” and “Association of AAM and 7q11.23 CNVs”, age refers to AAM; for “Differential Expression Analysis of STAG3L2 in 7q11.23 CNVs”, age refers to age at blood draw; for “Pituitary Volume Size Differences with 7q11.23 CNVs”, age refers to age at scan.

### Rare variant (7q11.23 CNVs) contributions to AAM

**Figure 2:**
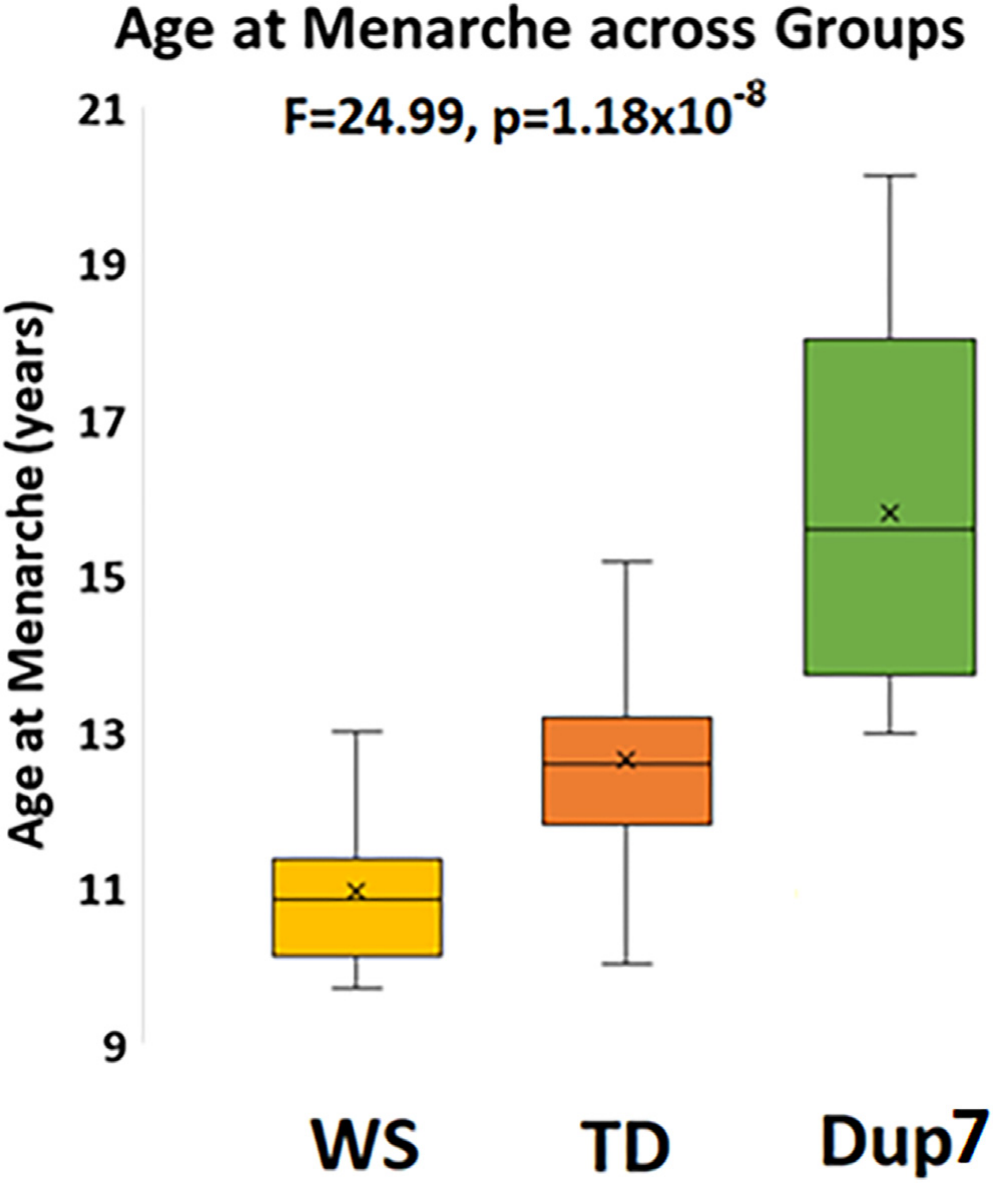
7q11.23 CNVs and differential pubertal timing AAM significantly differed across 7q11.23 CNV groups such that greater gene dosage was associated with older AAM (Average AAM: WS=10.96±1.1 years, TD=12.6±1.25, Dup7=15.8±2.7; F=24.99, η^2^=0.45, p=1.2x10^-8^). Post-hoc tukey’s HSD tests showed AAM in the WS group significantly differed from TDs (p=0.01) as did AAM of the Dup7 group compared to TDs (p=5.7x10^-7^). X’s represent mean of each group, lines represent median, boxes represent the range of the central 50% of data, and whiskers represent maximum and minimum values. Abbreviations: WS: Williams syndrome; Dup7: 7q11.23 Duplication syndrome; TD: typically-developing children.

Clinical observations have suggested that individuals with WS (hemideletions yielding one copy of ∼25 genes at chromosomal locus 7q11.23) have early puberty, whereas those with Dup7 (having three copies of these same genes) have delayed puberty. We sought to confirm these observations empirically and ascertained AAM through clinical interviews of eight girls with WS, 47 TD girls, and nine girls with Dup7 (Table 1). In our rare cohorts of people with 7q11.23 CNVs, we found that AAM significantly differed across copy number groups such that greater gene dosage was associated with older AAM (Average AAM: WS = 10.96 ± 1.1 years, TD = 12.6 ± 1.25, Dup7 = 15.8 ± 2.7; F = 24.99, η^2^ = 0.45, p = 1.2x10^−8^, Figure 2), with 7q11.23 copy number accounting for 45% of the variance in AAM. Post-hoc Tukey’s honest significant difference tests showed that AAM in the WS group was significantly earlier than was AAM in both TDs (p = 0.01) and Dup7 (p = 5.7x10^−7^) and AAM in the Dup7 group was significantly later than in TDs (p = 2.3x10^−8^).

Because common genetic and environmental factors could influence the timing of puberty, we also collected AAM data on typically developing full sibling girls of our participants with WS (8 siblings, AAM = 12.7 ± 1.1) and Dup7 (8 siblings, AAM = 12.2 ± 0.8). These sibling participants (who have the typical two copies of 7q11.23 genes) live and have grown up in the same households as the CNV participants. Therefore, their development could have been affected not only by similar genetic factors (outside of the 7q11.23 region), but also by similar environmental factors that may affect the timing of puberty, including nutrition and socioeconomic status. We found no AAM differences between the unrelated TD group and the 16 siblings of our patients (unrelated TD AAM 12.6 years, sibling AAM 12.45 years, F = 0.6, p = 0.5). Additionally, we repeated our analysis of AAM as a function of copy number status, substituting these siblings for unrelated TDs to carry out comparison with the two 7q11.23 CNV groups and confirmed the stepwise copy number effect on AAM controlling for these shared genetic and environmental factors (F = 17.8, η^2^ = 0.5, p = 8x10^−5^).

Because one individual with Dup7 included in this analysis was treated with growth hormone during childhood, we repeated the analysis testing for association of AAM with 7q11.23 copy number excluding this individual, and the results were largely unchanged and remained highly significant (F = 20.55, η^2^ = 0.41, p = 1.6x10^−7^).

### Pituitary volume in individuals with 7q11.23 CNVs

**Figure 3:**
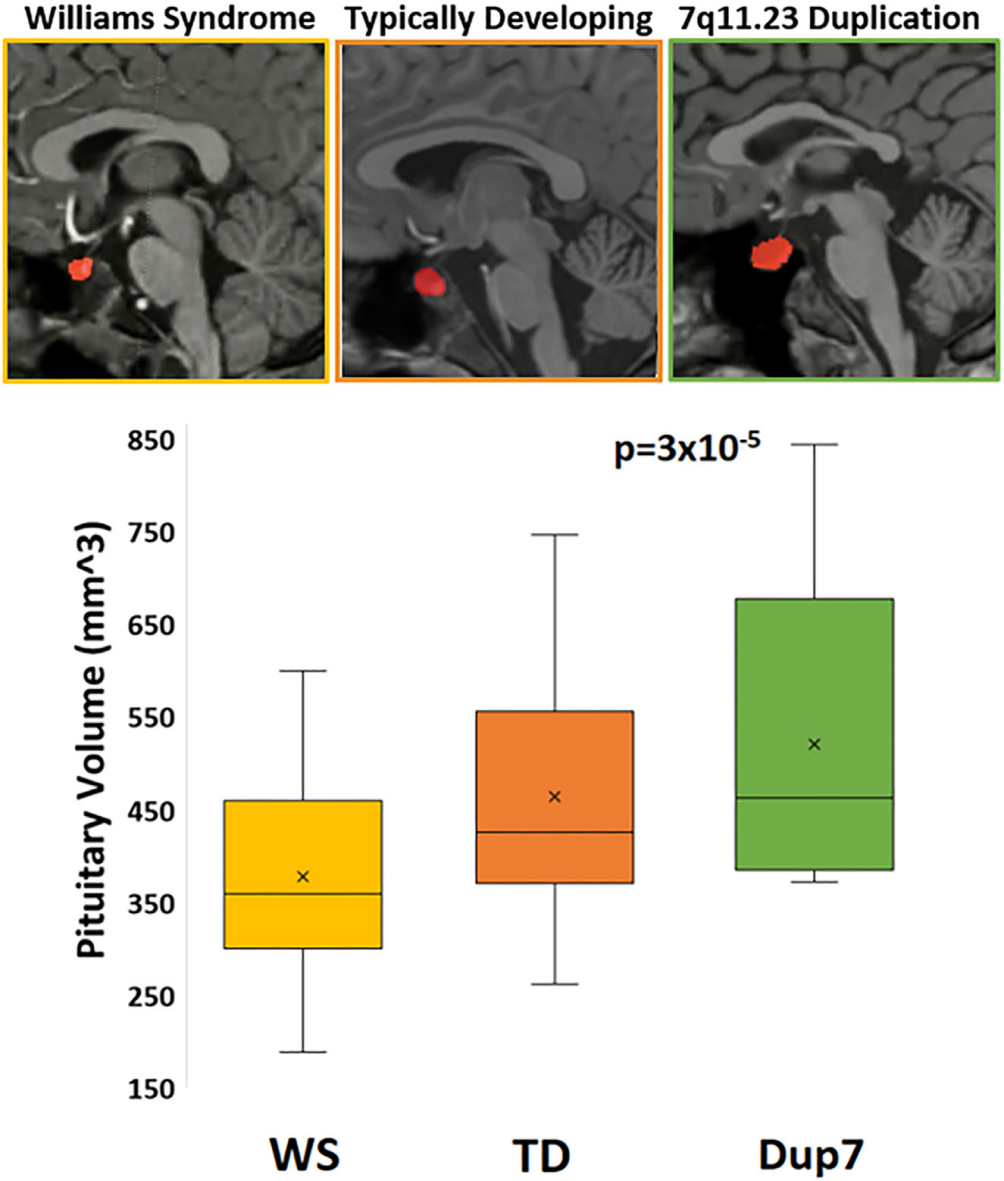
7q11.23 CNVs and differential pituitary volumes Pituitary size was significantly associated with 7q11.23 copy number, such that individuals with WS had the smallest pituitary glands while individuals with Dup7 had the largest (p=3x10^-5^). Top: Sample pituitary segmentations of a representative individual with WS (left), a TD participant (middle), and an individual with Dup7 (right). Bottom: Box plots of pituitary volumes as a function of CNV group (WS N=30, age=11.87±3.9 years; TD N=50, age=11.88±3.4 years; Dup7 N=16, age=12.51±2.8 years). X’s represent mean of each group, lines represent median, boxes represent the range of the central 50% of data, and whiskers represent maximum and minimum values. Abbreviations: WS: Williams syndrome; Dup7: 7q11.23 Duplication syndrome; TD: typically-developing children.

The pituitary gland is a key brain region in regulating endocrine function and is central to the HPG axis, producing a surge of hormones at the onset of puberty. Additionally, this region is also enriched with genes underlying AAM-related genetic loci,^23^ suggesting that the epidemiologic association between AAM and diseases later in life may be attributed, at least in part, to variability in exposures to sex hormones related to menarche. Prior evidence points to pituitary size increasing in puberty with potentially larger glands in individuals with early puberty.^41^^,^^42^ To test whether individuals with rare 7q11.23 CNVs, who (as aforementioned) showed differential timing of AAM, also have differential sizes of pituitary glands, raters, who were blinded to participants’ copy number status, manually segmented the pituitary from structural brain MRIs of 30 individuals with WS, 50 TD children and 16 individuals with Dup7, matched for age (Table 1). Total volume of the segmented region was calculated and compared across groups. Pituitary size was significantly associated with 7q11.23 copy number, such that individuals with WS had the smallest pituitary glands, while individuals with Dup7 had the largest (F = 11.7, η_p_^2^ = 0.2, p = 3x10^−5^, Figure 3). Post-hoc pairwise comparisons showed that the pituitary size of participants with WS was significantly smaller than both TDs (p = 1.1x10^−4^) and those with Dup7 (p = 4.5x10^−5^); TDs were nominally smaller than Dup7 (p = 0.17). The directionality of this across-group relationship was opposite to that expected, since as aforementioned, individuals with precocious puberty have larger pituitary volumes,^41^^,^^42^ but here, individuals with Dup7, who have later AAM, had larger pituitary volumes. In reviewing the images after raters blinded to copy number status had manually performed pituitary segmentation, it was noted that glands of several individuals with Dup7 had a more cystic appearance, potentially suggesting a buildup of unreleased hormone.^44^^,^^45^

**Figure 4:**
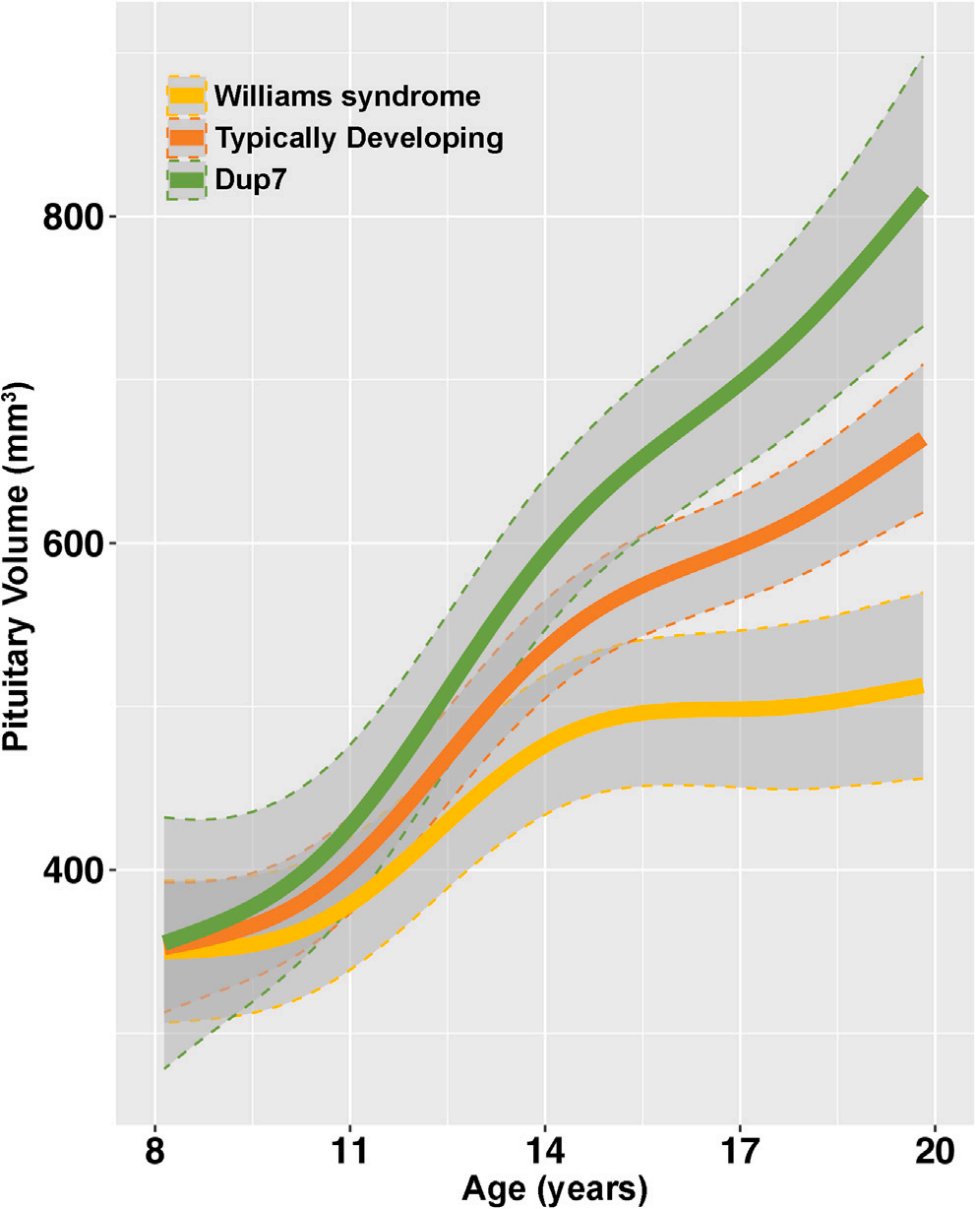
Developmental trajectories of 226 longitudinally acquired pituitary volumes (in cubic millimeters) graphed by 7q11.23 copy number status Pituitary volumes from T1-weighted MRIs of 30 participants with Williams syndrome ([WS], 87 visits), 50 typically developing children ([TD], 102 visits), and 16 participants with 7q11.23 Duplication ([Dup7], 37 visits) over 1–5 visits for each participant across ages 8–20 years. The three developmental trajectory curves (WS, yellow; TD, orange; and Dup7, green) were longitudinally calculated with mixed-effects spline modeling; gray ribbons depict 95% confidence intervals for each group’s curve. There were significant age (p = 2x10^−16^) and group (p = 2x10^−4^) effects and an age-by-group interaction (p = 2x10^−6^): pituitary volumes of the three groups were similar until after the start of puberty, but then began to diverge, with largest pituitary volumes in individuals with Dup7 and smallest volumes in WS, a pattern that continued throughout the studied age range; volume increased at a faster rate in the Dup7 group.

Next, to examine developmental patterns of pituitary volume longitudinally within 7q11.23 copy number groups, investigators, again blind to diagnosis, segmented pituitaries from 226 longitudinal visits of these same participants, many of whom return approximately every two years. This analysis included scans from 87 visits of 30 participants with WS, 102 visits of 50 TDs, and 37 visits of 16 participants with Dup7. There were significant age (p = 2x10^−16^) and group (p = 2x10^−4^) effects and an age-by-group interaction (F = 10.02, p = 2.5x10^−6^), wherein pituitary volumes of the three groups were similar after the start of puberty, but then begin to diverge, and the reduced volumes in WS and the increased volumes in Dup7 become apparent (Figure 4).

Additionally, to ensure that these analyses were not driven by treatment with any hormonal medications, we performed sensitivity analyses for both the cross-sectional and longitudinal assessment of pituitary volumes after excluding participants who had any history of receiving treatment with hormonal medications. The results of these sensitivity analyses were largely unchanged from those of the primary analyses: for the cross-sectional analysis (22 participants with WS, 50 TDs, and 15 participants with Dup7; F = 8.22, η_p_^2^ = 0.17, p = 5.5x10^−4^; and for the longitudinal age-by-group interaction F = 6.02, p = 4.5x10^−4^, based on 186 total visits.

### 7q11.23 genetic signals impacting AAM

**Figure 5:**
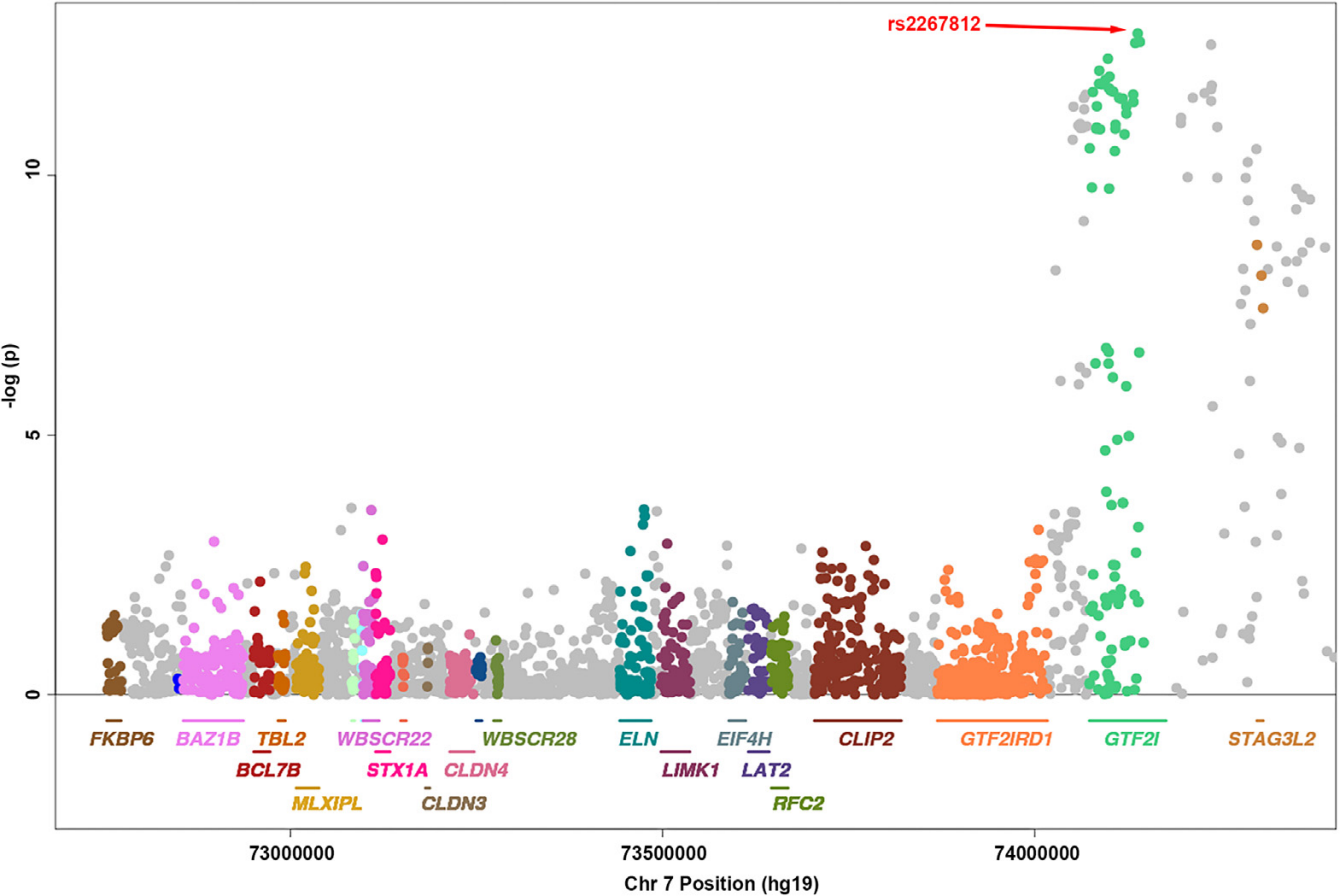
SNP associations with AAM in the expanded 7q11.23 region A “mini-Manhattan” plot of SNP associations with AAM across the expanded WS critical region. One genome-wide significant signal spanning the telomeric end of the WS critical region was found, extending into the low-copy repeat region that telomerically flanks the WS 7q11.23 locus (peak SNP rs2267812, p = 1.7x10^−17^). Abbreviations: SNP: single nucleotide polymorphism; AAM: age at menarche; WS: Williams syndrome.

**Figure 6:**
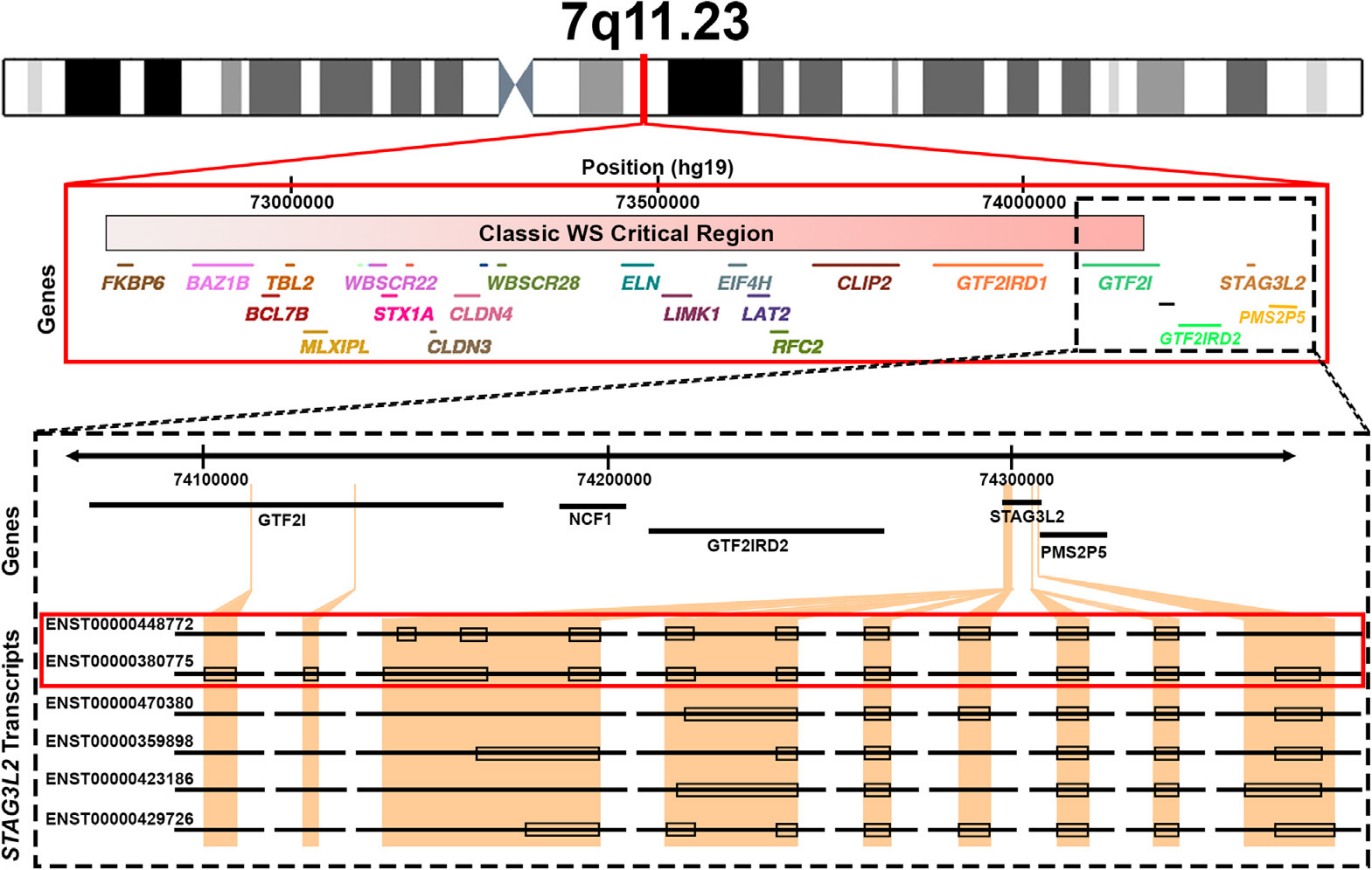
*STAG3L2* alternate transcripts Figure highlights the genes in the WS region and *STAG3L2* transcripts significantly related to AAM-associated SNP rs2267812. Top shows WS critical region at the 7q11.23 locus; colored lines and gene names indicate affected individual genes, and the red gradient bar represents the classic WS hemideletion/Dup7 duplication. Bottom shows locations of introns for *STAG3L2* transcripts as reported in Ensembl (http://www.ensembl.org). The first two transcripts, highlighted by the red box, represent those showing significant eQTLs with rs2267812 in data derived from postmortem brains of healthy individuals from the brainseq database (http://eqtl.brainseq.org/phase1/eqtl/; ENST00000448772 and ENST00000380775, p_bonf_ = 0.002 and p_bonf_ = 0.01, respectively. Importantly, the ENST00000380775 transcript spans 194kb, nearly the entire identified significant region, and includes the telomeric portion of the 7q11.23 region hemideleted in WS. In contrast, the ENST00000448772 transcript lies exclusively in the flanking region and is not typically involved in the classical WS deletion.

To determine which, if any, of the 389 genetic signals previously reported to be significantly associated with AAM in a large-scale GWAS^23^ are located at the 7q11.23 locus, we referenced the reported summary statistics from that study. One genome-wide significant signal spanning the telomeric end of the WS critical region was identified, extending into the low-copy repeat region flanking the WS 7q11.23 locus (peak SNP rs2267812, p = 1.7x10^−17^, Figure 5). To further characterize this significant locus, we searched for eQTLs in data derived from postmortem brains of healthy individuals from the Brainseq database (http://eqtl.brainseq.org/phase1/eqtl/). Though this peak SNP, rs2267812, is located within the *GTF2I* gene, we found that its variation was most significantly associated with expression of two transcripts of the *STAG3L2* gene (ENST00000448772 and ENST00000380775, p_bonf_ = 0.002 and p_bonf_ = 0.01, respectively), suggesting that the latter gene may be responsible for the association of this locus with AAM. Importantly, the ENST00000380775 transcript of *STAG3L2* spans 194kb, nearly the entire locus seen in Figure 5 and includes an exon underlying the *GTF2I* gene in the telomeric portion of the 7q11.23 region hemideleted in WS (Figure 6). In contrast, the ENST00000448772 transcript lies exclusively in the flanking region that is not typically involved in the classic WS hemideletion (Figure 6), further emphasizing the importance of ENST00000380775 for this work.

**Figure 7:**
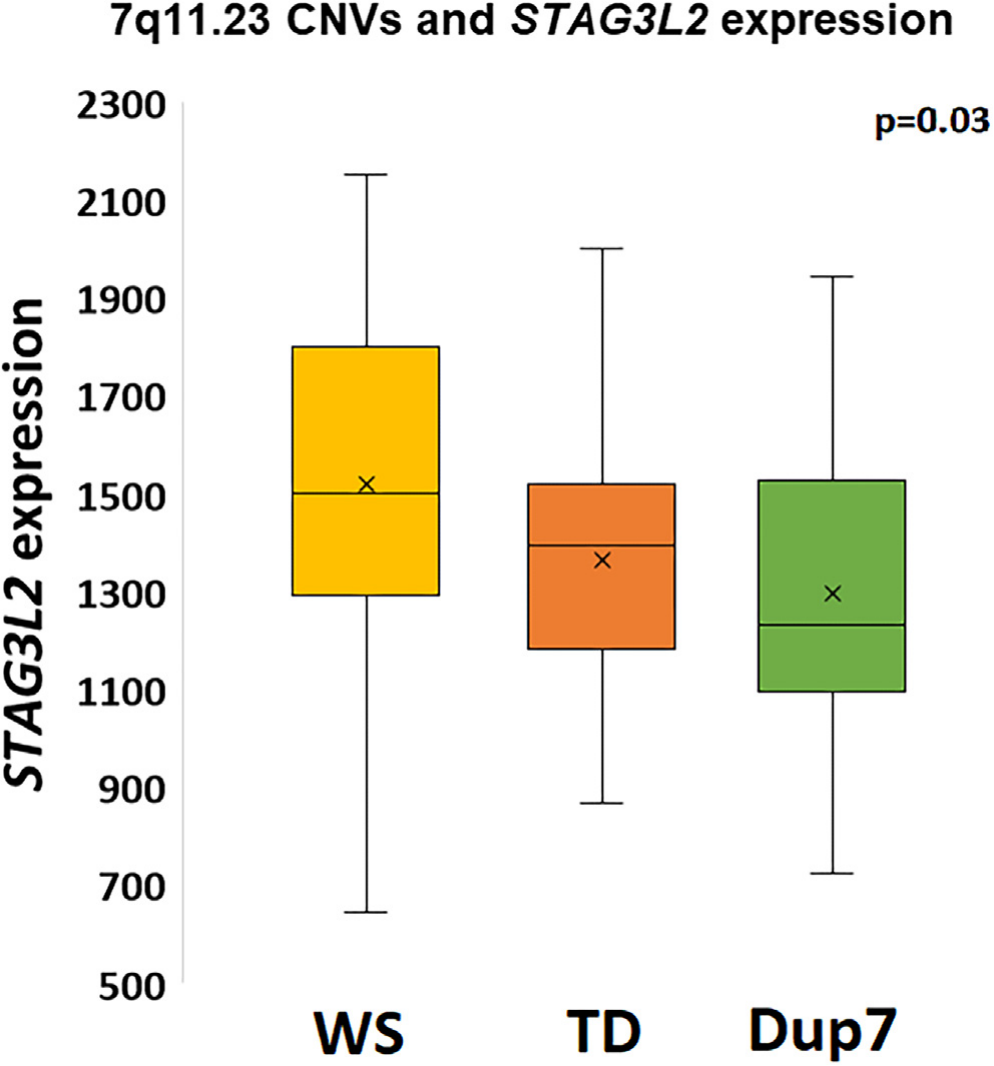
7q11.23 CNVs and *STAG3L2* expression RNASeq analysis showed that expression of *STAG3L2* was highest in individuals with WS (who have hemideletions) and lowest in individuals with Dup7 (who have an extra copy at 7q11.23, p = 0.03). Y axis shows normalized *STAG3L2* counts from DESEQ2. X’s represent mean of each group, lines represent median, boxes represent the range of the central 50% of data, and whiskers represent maximum and minimum values. Abbreviations: WS: Williams syndrome; Dup7: 7q11.23 Duplication syndrome; TD: typically developing children.

Though one exon of the ENST00000380775 transcript of *STAG3L2* extends into the classic WS critical region, the typical reporting of this gene lies outside of the WS locus, in the flanking low-copy repeat region (Figure 6, bottom). Therefore, to test whether individuals with WS and Dup7 have altered expression of *STAG3L2*, we analyzed RNASeq data from blood lymphocytes of 23 children with WS, 40 TD children, and 13 children with Dup7 (Table 1), and found that expression of *STAG3L2* was related to CNV dosage (p = 0.03; Figure 7). Post-hoc t-tests showed that individuals with WS had greater *STAG3L2* expression than both TDs (p = 0.03) and individuals with Dup7 (p = 0.04) but the greater expression in TDs compared to Dup7 was not significant (p = 0.2). Surprisingly, the directionality of this finding was such that expression of *STAG3L2* was highest in individuals with WS (who have hemideletions) and lowest in individuals with Dup7 (who have an extra copy of the ∼25 genes in the WS 7q11.23 locus), suggesting that hemideletion of this region may include the removal of regulatory element(s) that inhibit *STAG3L2* transcription. We also examined RNAseq data regarding the expression of the other *STAG3*-like genes (which are not located in the 7q11.23 CNV region) and found no associations between 7q11.23 copy number status and expression of these genes. These include *STAG3* (p = 0.98), *STAG3L1* (p = 0.2), *STAG3L3* (p = 0.96), *STAG3L4* (p = 0.42), *STAG3L5P* (p = 0.99), and *STAG3L5P-PVRIG2P-PILRB* (p = 0.98).

## Discussion

Consistent with prior clinical observations, we empirically confirmed that individuals with 7q11.23 CNVs have differential pubertal timing, such that greater gene dosage (i.e., in participants with Dup7) was associated with older AAM, whereas hemideletion (i.e., in WS) was associated with earlier pubertal timing. In support of our focus on these rare neurodevelopmental conditions in order to better understand genetic sources of AAM variability, we found that CNV status explained approximately 45% of our sample’s total variance in AAM, reflecting both the wide range of AAM in our CNV cohorts and the strength of the highly penetrant CNV effect. Additionally, our observation is likely an underestimation of the actual CNV effect, as the girls with WS who were treated with hormonal medication to delay their early onset of puberty were not able to be included in this analysis as their AAM was pharmacologically altered. In contrast to this robust copy number effect, the cumulative effects of GWAS-identified common genetic variation, while present, accounted for ∼9% of the variance in AAM in our reference sample of TD children, which importantly, is similar to the previously reported ∼7.4% of the population variance estimated from prior large-scale GWAS data.^23^

Though there is a significant genetic component driving AAM, environmental factors, including nutrition and socioeconomic status, could also affect development. Our analysis of AAM comparing patients with 7q11.23 CNVs to full siblings (who have the typical two copies of 7q11.23 genes and live and have grown up in the same households as the patients) was highly significant. In fact, the effect size estimate actually increased (from η^2^ = 0.45 to 0.5) despite a substantially smaller cohort of siblings than there were unrelated TD individuals with 2 copies of 7q11.23 (Ns = 47 TDs versus 16 sibling girls), suggesting that taking the shared genetic (outside of the 7q11.23 region) and environmental factors into consideration brought the observed copy number effect into even clearer focus.

In addition to AAM, pituitary size was also associated with 7q11.23 copy number, such that individuals with WS had the smallest pituitary glands while individuals with Dup7 had the largest. This result indicates that pituitary development is likely subject to genetic regulation originating, at least in part, from within the 7q11.23 locus. The pituitary gland, a central endocrine hub governing sex hormone homeostasis and playing a crucial role in the pubertal transition, shows volumetric changes associated with sexual development and sex hormone milieu^46^^,^^47^ and is enriched for AAM-associated gene transcripts.^23^ Moreover, SNPs near genes involved in hormone synthesis, bioactivity (e.g., *ESR1*, *PGR*), and pituitary function (e.g., *TACR3*, *LGPR4*) have been found to be associated with menarche timing.^48^ However, the directionality of the association between pituitary size and 7q11.23 CNVs—individuals with WS having smaller pituitary volumes (and earlier puberty) and individuals with Dup7 having larger pituitaries (and later puberty)—suggests that the molecular mechanisms driving earlier puberty in WS may be different from those for clinical disorders associated with formal diagnoses of central precocious puberty, wherein pituitary volumes have been found to be enlarged.^41^^,^^42^ Additionally, our qualitative observation of more cystic-appearing pituitary glands in individuals with Dup7 will require further investigation, including determining whether this observation might indicate a buildup of unreleased hormone. Regardless, because of the pituitary’s important role in pubertal development and concomitant morphological changes mirroring pubertal timing abnormalities, it is unlikely that the parallel 7q11.23 CNV associations with both AAM and pituitary size are independent phenomena. Future work will be needed to delineate potential biological pathways leading from specific WS critical region gene hemideletion/duplication to pituitary structural and functional change resulting in AAM alterations.

Given the results we obtained from combining large-scale GWAS-level information with incisive studies of individuals with rare 7q11.23 CNVs, one candidate gene deserving of such investigation is *STAG3L2*. We identified one genome-wide significant signal from the Day et al., findings^23^ that spans the telomeric end of the 7q11.23 CNV region, extending into the low-copy repeat region flanking the WS locus (peak SNP rs2267812). RNASeq data from postmortem brains suggest that, though the peak SNP is physically located in an intron of the *GTF2I* gene, variation at rs2267812 results in differential expression of a different gene, *STAG3L2*. This gene is ubiquitously expressed, particularly in testes, ovaries and pituitary,^37^ and is similar in structure to at least five other “*STAG3*-like” genes, all of which lie outside the 7q11.23 WS critical region in other low copy repeat regions on chromosome 7.

Interestingly, the existence of these multiple “*STAG3*-like” genes is believed to be due to segmental duplications that first occurred in a common ancestor to all hominds after diverging from macacques 12–19 Mya, though the emergence of the *STAG3L2* gene of interest here likely occurred during a much more recent segmental duplication in evolution, estimated to have occurred 2.55–2.89 Mya.^49^ Though little is known about these “*STAG3*-like” genes, including the *STAG3L2* gene of focus here, the ancestral *STAG3* gene, which lies telomeric to the WS critical region, at chromosomal locus 7q22, is a cohesion complex component known to be involved in meiotic chromosome pairing^50^^,^^51^ and is particularly integral to the sistering of chromatid arms during meiosis I.^52^ Further, mutations in this ancestral *STAG3* gene have been implicated in disorders of reproductive functions in both men (spermatogenic impairment)^53^^,^^54^^,^^55^ and women (primary ovarian insufficiency).^51^^,^^53^^,^^56^^,^^57^^,^^58^^,^^59^^,^^60^ Indeed, the findings reported here suggest that the structurally similar *STAG3L2* gene is also an important mediator in endocrine function and has the potential to also influence the onset of puberty.

The observation from our blood lymphocyte RNASeq analyses of individuals with 7q11.23 CNVs that an increase in *STAG3L2* RNA expression is linked to WS is surprising, as one might expect hemideletion to result in a decrease in expression. This unexpected result suggests that hemideletion of the WS critical region may lead to the removal of regulatory elements that normally inhibit transcription. These findings are particularly intriguing in light of the reported links between *STAG3* loss-of-function mutations and primary ovarian insufficiency in women, which is also characterized by delayed puberty. This association the ancestral *STAG3* gene with pubertal timing is consistent with our observations in individuals with Dup7, in whom *STAG3L2* expression is decreased and puberty is delayed, versus in WS, where *STAG3L2* expression is increased and puberty is early. Thus, it seems plausible that the altered *STAG3L2* expression observed in WS and Dup7 and altered *STAG3L2* expression associated with common variation linked AAM (i.e., rs2267812) could reflect a unitary genetic mechanism impacting the timing of puberty. Understanding the precise mechanisms underlying these associations will require further research, but these findings may ultimately shed light on the complex interplay between genetics and pubertal timing, and the role of *STAG3L2* in these processes.

While much of our focus on the GWAS signal studied here has been on the *STAG3L2* gene, the physical location of the peak SNP in the *GTF2I* gene offers a possible alternative candidate for the gene of interest underlying this locus. However, given the associations between *STAG3* and ovarian insufficiency, the eQTL linking rs2267812 with *STAG3L2* expression in the postmortem brain, and the alternative *STAG3L2* transcript that spans the entire significant GWAS locus, *STAG3L2* appears to be the more likely candidate responsible for the differential impacts on pubertal timing observed in individuals with WS and Dup7. Nonetheless, *GTF2I* codes for a general transcription factor (TFII-I) that regulates methylation of genes outside of the WS critical region^61^ and its targets include regulators of phosphorylation and WNT signaling.^62^ Additionally, variation in this gene has been associated with the hypersocial phenotype seen in WS^63^ and recent evidence has linked this gene to oligodendrocyte function and myelination in the human and murine brain.^64^ Therefore, this important gene warrants further study, but may be the less likely candidate for the pubertal effects reported here.

Overall, the findings presented here provide valuable insights into the genetic mechanisms underlying the timing of puberty and the role of the 7q11.23 locus, particularly the *STAG3L2* gene, in this pivotal developmental process. Specifically, the study confirmed that individuals harboring AAM-associated common polymorphisms and those with 7q11.23 CNVs have differential pubertal timing, with relatively modest versus large effect sizes, respectively. Furthermore, alongside AAM, the size of the pituitary may be modulated by 7q11.23 CNVs and merits investigation as a potential neural substrate for relevant pubertal events. Additionally, we suggest that *STAG3L2* may yoke both rare CNV and common-SNP findings in this arena, potentially representing a key mediator of altered pubertal onsets in 7q11.23 CNV syndromes that may also generalize to normative variation in the timing of puberty. Further research will be necessary in order to understand the precise mechanisms underlying these associations, but collectively, the present data provide valuable insights into, and pathways for further discovery of, the complex interplay between genetics and puberty timing. They also present a potential avenue to investigate the adverse health consequences associated with the timing of puberty, which present substantial public health concerns.

### Limitations of the study

Several caveats warrant mention regarding the present work. First, because of the relatively small sample sizes of these rare CNVs, we are unable to directly assess the effects of race or early stress, which, in addition to genetics, might influence AAM. Also, there is relatively limited data assessing age at voice breaking in boys, a corresponding pubertal milestone in males that shares many similar genetic signals with AAM in girls.^23^ Though there may be multiple reasons for these limited data, including the fact that our cohort of individuals with WS has a sampling bias toward girls, the timing voice breaking in boys has proved to be more difficult to ascertain than AAM. In our experience, individuals and parents tend to remember the exact date of the discrete event of menarche in girls but are less likely to remember the approximate date of the relatively slower process of voice breaking in boys. Lastly, mechanistic interpretation of these findings related to sex steroids is limited by the lack of information regarding circulating hormone levels in these participants. Therefore, the presence of any such association with these data will require further study.

## STAR★Methods

### Key resources table

**Table:** 

REAGENT or RESOURCE	SOURCE	IDENTIFIER
**Deposited data**		

GWAS summary statistics for AAM from Day et al. 2017	https://www.reprogen.org/	Menarche_1KG_NatGen2017_WebsiteUpload.zip

**Software and algorithms**		

Plink2	https://www.cog-genomics.org/plink/2.0	NA
IBM SPSS Statistics v29.0.0.0	https://www.ibm.com/spss	NA
Gamm4 R package	https://cran.r-project.org/web/packages/gamm4	NA
STAR version 2.6.1	https://github.com/alexdobin/STAR	NA
DESeq2	https://bioconductor.org/packages/release/bioc/html/DESeq2.html	NA
QoRTS version 1.3.6	https://github.com/hartleys/QoRTs/releases/tag/v1.3.6	NA

### Resource availability

#### Lead contact

Further information and requests for resources and reagents should be directed to the lead contact, Karen Berman (bermank@mail.nih.gov).

#### Materials availability

This study did not generate new unique reagents.

#### Data and code availability

• Data: GWAS summary statistics used for polygenic scoring were obtained from the supplementary data associated with the cited Day et al. publication.^23^ Some data from the NIMH Intramural Research Program study defining the brain phenotype of children with WS and Dup7 are not publicly available due to institutional (IRB) restrictions. Data from the NIMH Intramural Longitudinal Study of the Endocrine and Neurobiological Events Accompanying Puberty will be publicly shared as permitted via IRB approval and participant consents.
• Code: This paper did not report original code. Software packages used are reported in the key resources table.
• All Other Items: Any additional information required to reanalyze the data reported in this paper is available from the lead author upon request.

### Experimental model and study participant details

Participants were recruited as part of our long-term, longitudinal NIMH Intramural Research protocol, Defining the Brain Phenotype of Children with WS and Dup7, and were studied at the NIH Clinical Center (Protocol #10-*M*-0112, NCT01132885, ZIAMH002863). Additionally, some TD girls were recruited from the NIMH Intramural Longitudinal Study of the Endocrine and Neurobiological Events Accompanying Puberty (Protocol #11-*M*-0251, NCT01434368, ZIAMH002717/ZIAMH002933). Study procedures were approved by the NIH IRB. Parents of minor participants provided written informed consent and children provided assent. All participants were in good physical health, based on physical examination and a review of available medical records, had IQs in the low-normal to normal range based on neuropsychological testing, and had no significant morphological abnormalities on clinical brain MRI as well as intact cerebral vasculature, based on a radiologist’s reading of MR images and MR angiography, respectively. Finally, no WS or Dup7 participants reported thyroid issues and no participants with Dup7 were placed on medications to alter pubertal timing. Demographic characteristics, including age and sex, are reported in Table 1, separately for each analysis reported below.

### Method details

#### Analysis of AAM polygenic scores in TD girls

Blood samples were collected from participants and genotyped using Illumina HumanOMNI-5M SNP chips. Data were phased using Shapeit and imputed using Impute2 software, as previously reported.^65^ Polygenic scores (PGSs) for AAM were calculated with PLINK 2’s “score” function,^66^ based on previously reported GWAS summary statistics,^23^ using 10 p value thresholds (ranging from 5 × 10^−8^ to 1.0). The polygenic signal was concentrated into a single score through principal component analysis and the first principal component was carried forward for further analyses, based on previously described methods.^67^^,^^68^ The relation between the resulting PGS-AAM scores and clinician-ascertained AAM was determined using Pearson correlation in SPSS.

#### Association of AAM and 7q11.23 CNVs

AAM was ascertained, as above, through clinical interview of participants and their parents, including 17 girls with 7q11.23 CNVs: eight with WS, nine with Dup7, and 47 typically developing (TD) girls. AAM was also ascertained from 8 siblings of individuals with WS and 8 siblings of individuals with Dup7. Seven additional girls with WS had been excluded from analysis as they were treated with hormonal medications to pharmacologically delay the onset of puberty. No participants with Dup7 received hormonal treatment to alter the timing of puberty, and therefore none were excluded for this reason. A one-way ANOVA was used to calculate group differences in AAM in SPSS across WS, TD, and Dup7 participants. Post-hoc Tukey’s honest significant difference tests were performed between each group to assess whether AAM significantly differed between WS and TD groups and between Dup7 and TD groups. Additionally, a one-way ANOVA was also used to examine other common genetic and environment factors that may influence the timing of puberty across WS siblings, TD, and Dup7 siblings.

#### Pituitary Volume Size Differences with 7q11.23 CNVs

For age-matched groups of 30 individuals with WS, 50 TD children, and 16 individuals with Dup7, investigators blinded to diagnostic group determined the volume of the pituitary by manual segmentation on T1-weighted ME-MPRAGE structural scans (TR/TE: 10.5/1.8 ms, Flip Angle: 7°, voxel size: 1 × 1 × 1mm, 176 slices) collected on a GE 3T MRI scanner. Group differences in pituitary volumes across 7q11.23 copy number groups were assessed in SPSS using a general linear model, controlling for age at scan and sex. Post-hoc pairwise comparisons of estimated marginal means were also reported from the general linear model in SPSS. To further examine the developmental patterns of the pituitary volume across 7q11.23 CNV, 226 longitudinal visits (1–5 visits per participant, age range: 5–24 years old) of these same participants (all children returned approximately every two years for the study; WS: N = 30, 87 visits; TD: N = 50, 102 visits; Dup7: N = 16, 37 visits) were used to evaluate the development of pituitary size. R’s gamm4 statistical package was used to carry out a mixed-effects penalized-spline analysis to model age-dependent developmental trajectories within these three diagnoses groups.

#### Differential expression analysis of STAG3L2 in 7q11.23 CNVs

RNA was extracted from lymphocyte cell lines for 23 children with WS, 40 TD children, and 13 children with Dup7, and RNA sequencing was performed at the NIH Intramural Sequencing Center. Stranded Poly-A selected mRNA libraries were constructed from 1 μg total RNA for each sample using the TruSeq Stranded mRNA Kit (Illumina) according to the manufacturer’s instructions. Amplification was performed using 10 cycles to minimize over-amplified product. Unique dual-indexed barcode adapters were applied to each library. Libraries were pooled in an equimolar ratio for sequencing. The pooled libraries were sequenced on an S4 flow cell on a NovaSeq 6000 using version 1.0 chemistry to achieve a minimum of 49 million 150 base read pairs. The data were processed using RTA version 3.4.4. Alignment of resulting RNASeq fastq files was performed using STAR version 2.6.1 to the GRCh37 genome build. Aligned reads were further processed using QoRTs version 1.3.6 and expression values of *STAG3L2* were analyzed using DESeq2 to test for stepwise group differences. Post-hoc pairwise t-tests of extracted normalized count values were conducted in SPSS to test for differences in *STAG3L2* expression between groups.

### Quantification and statistical analysis

For the analysis of AAM in TD children, the relation between PGS-AAM scores and clinician-ascertained AAM was determined using Pearson correlation in SPSS. For the analyses of AAM in 7q11.23 CNVs, one-way ANOVAs were used to calculate group differences in AAM in SPSS, and post-hoc Tukey’s honest significant difference tests were performed. For the cross-sectional analyses of pituitary volume, a general linear model was performed in SPSS, controlling for age at scan and sex. For the longitudinal pituitary volume data, mixed-effects penalized-spline modeling was analyzed using R’s gamm4 package. For the differential expression analysis, expression values were analyzed using DESeq2 to test for stepwise group differences and post-hoc pairwise t-tests of extracted normalized count values were conducted in SPSS.

### Additional resources

This work was is part of two clinical trials, NCT01434368 and NCT01132885.
